# Supplementary material for: Pre-operative kidney biomarkers and risks for death, cardiovascular and chronic kidney disease events after cardiac surgery: the TRIBE-AKI study
Source: J Cardiothorac Surg. 2022 Dec 25;17:338. doi: 10.1186/s13019-022-02066-4 (PMC9790121; doi:10.1186/s13019-022-02066-4)
Supplement: Supplementary file 1 — Additional file 1. Table S1. Patient characteristics stratified by sTNFR2 and KIM-1 tertile in TRIBE-AKI. Table S2. Biomarker Concentrations by all-cause mortality, cardiovascular events, chronic kidney disease events (Median [IQR] values are presented in each cell). Table S3. Assessment of the interaction by diabetes mellitus on the association between pre-operative biomarkers and long-term outcomes in TRIBE-AKI (Adjusted for recipient Age (per year), recipient gender, white race (Yes/No), non-elective surgery, surgery type, pre-op eGFR, diabetes, hypertension, CHF, history of MI, pre-op urine albumin and urine creatinine, clinical site). Table S4. Pre-operative patient characteristics stratified by availability of CKD outcome assessment in follow-up [file 13019_2022_2066_MOESM1_ESM.docx]

**Supplemental Table 1.** Patient characteristics stratified by sTNFR2 and KIM-1 tertile in TRIBE-AKI

|  | **sTNFR2** | | | | **KIM-1** | | | | |
| --- | --- | --- | --- | --- | --- | --- | --- | --- | --- |
| **Characteristic** | **Pre-op Tertile 1**  **(24-4284) (n=464)** | **Pre-op Tertile 2**  **(4290-5918) (n=465)** | **Pre-op Tertile 3**  **(5935-100000) (n=464)** | **P-value** | **Pre-op Tertile 1**  **(42-205) (n=464)** | **Pre-op Tertile 2**  **(205-290) (n=465)** | **Pre-op Tertile 3**  **(290-8600) (n=464)** | | **P-value** |
| Age, mean (at time of surgery) | 69.5 ± 10.2 | 72.3 ± 9.7 | 73.7 ± 8.8 | <0.001 | 69.5 ± 10.8 | 73.5 ± 8.7 | 72.5 ± 9.1 | <0.001 | |
| White Race | 436 (94.0%) | 444 (95.5%) | 432 (93.1%) | 0.29 | 437 (94.2%) | 445 (95.7%) | 430 (92.7%) | 0.1 | |
| Male sex | 349 (75.2%) | 310 (66.7%) | 304 (65.5%) | 0.002 | 325 (70.0%) | 321 (69.0%) | 317 (68.3%) | 0.9 | |
| Diabetes | 166 (35.8%) | 174 (37.4%) | 199 (42.9%) | 0.07 | 144 (31.0%) | 160 (34.4%) | 235 (50.6%) | <0.001 | |
| Hypertension | 348 (75.0%) | 372 (80.0%) | 392 (84.5%) | 0.002 | 347 (74.8%) | 377 (81.1%) | 388 (83.6%) | 0.003 | |
| EF <35% or grade 3 or 4 LV dysfunction | 43 (9.3%) | 45 (9.7%) | 48 (10.3%) | 0.86 | 42 (9.1%) | 45 (9.7%) | 49 (10.6%) | 0.7 | |
| Myocardial infarction | 102 (22.0%) | 117 (25.2%) | 138 (29.7%) | 0.044 | 116 (25.0%) | 109 (23.4%) | 132 (28.4%) | 0.4 | |
| Congestive heart failure | 77 (16.6%) | 95 (20.4%) | 142 (30.6%) | <0.001 | 85 (18.3%) | 99 (21.3%) | 130 (28.0%) | 0.001 | |
| Mean Preoperative serum Cr, mg/dL | 0.9 ± 0.2 | 1.0 ± 0.2 | 1.3 ± 0.4 | <0.001 | 0.9 ± 0.2 | 1.0 ± 0.3 | 1.2 ± 0.4 | <0.001 | |
| Preoperative eGFR, mL/min/1.73m^2^  (mean ± SD) | 79.6 ± 14.6 | 70.2 ± 15.7 | 54.8 ± 18.3 | <0.001 | 75.0 ± 16.3 | 68.9 ± 17.7 | 60.6 ± 20.6 | <0.001 | |
| CKD stage ≥ 3 | 41 (8.8%) | 132 (28.4%) | 292 (62.9%) | <0.001 | 87 (18.7%) | 139 (29.9%) | 239 (51.5%) | <0.001 | |
| Urine Albumin to Cr ratio, mg/g (mean±SD) | 0.04 ± 0.1 | 0.1 ± 0.1 | 0.3 ± 2.5 | 0.04 | 0.03 ± 0.1 | 0.2 ± 0.1 | 0.3 ± 2.5 | 0.03 | |
| Surgery type |  |  |  | 0.13 |  |  |  | 0.02 | |
| CABG and valve | 85 (18.3%) | 108 (23.2%) | 117 (25.2%) |  | 90 (19.4%) | 99 (21.3%) | 121 (26.1%) |  | |
| Other | 9 (1.9%) | 4 (0.9%) | 6 (1.3%) |  | 2 (0.4%) | 12 (2.6%) | 5 (1.1%) |  | |
| CABG only | 242 (52.2%) | 230 (49.5%) | 207 (44.6%) |  | 247 (53.2%) | 216 (46.5%) | 216 (46.6%) |  | |
| Valve only | 128 (27.6%) | 123 (26.5%) | 134 (28.9%) |  | 125 (26.9%) | 138 (29.7%) | 122 (26.3%) |  | |
| Nonelective surgery | 405 (87.3%) | 402 (86.5%) | 356 (76.7%) | <0.001 | 389 (83.8%) | 401 (86.2%) | 373 (80.4%) | 0.05 | |

**Supplementary Table 2.** Biomarker Concentrations by each outcome.

|  | **All-cause mortality** | | | **Cardiovascular event** | | | **Chronic Kidney Disease events** | | |
| --- | --- | --- | --- | --- | --- | --- | --- | --- | --- |
|  | **Died** | **Survived** | **P-value** | **Yes** | **No** | **P-value** | **Yes** | **No** | **P-value** |
| **sTNFR1 (pg/mL)** | 8937  (6696-12708) | 7038  (5740-9088) | <0.001 | 7540  (6278-9965) | 7036  (5710-9082) | <0.001 | 8566  (6508-12226) | 7303  (5939-9774) | 0.002 |
| **sTNFR2 (pg/mL)** | 5817  (4539-8418) | 4765  (3764-6044) | <0.001 | 5040  (3957-6728) | 4889  (3792-6343) | <0.001 | 5711  (4188-8113) | 4927  (3873-6456) | 0.06 |
| **KIM-1 (pg/mL)** | 284.1  (228.2-379.6) | 223.8  (173.1-296.5) | <0.001 | 269.6  (204.9-335.2) | 227.0  (176.6-299.4) | <0.001 | 276.3  (215.2-371.5) | 239.4  (180.9-316.3) | <0.001 |

Median (IQR) values are presented in each cell.

**Supplementary Table 3.** Assessment of the interaction by diabetes mellitus on the association between pre-operative biomarkers and long-term outcomes in TRIBE-AKI

| **Biomarker (log)** | **Interaction variable** | **All-Cause Mortality** | | | **Cardiovascular Events** | | | **Chronic Kidney Disease Events** | |
| --- | --- | --- | --- | --- | --- | --- | --- | --- | --- |
|  |  | **Adjusted*** **HR (95% CI)** | **Interaction p-value** | **Adjusted*** **HR (95% CI)** | | **Interaction p-value** | **Adjusted*** **HR (95% CI)** | | **Interaction**  **p-value** |
| **sTNFR1** | DM No | 2.3 (1.8, 3.7) | 0.5 | 1.9 (1.1, 3.1) | | 0.9 | 1.9 (1.1, 3.2) | | 0.2 |
|  | DM Yes | 3.6 (2.4, 5.5) |  | 2.3 (1.3, 3.9) | |  | 2.9 (1.8, 4.7) | |  |
| **sTNFR2** | DM No | 2.1 (1.6, 2.8) | 0.8 | 1.9 (1.3, 2.8) | | 0.5 | 1.5 (0.9, 2.5) | | 0.09 |
|  | DM Yes | 3.5 (2.2, 5.5) |  | 1.8 (1.0, 3.2) | |  | 2.8 (1.6, 4.9) | |  |
| **KIM-1** | DM No | 2.6 (1.8, 3.7 | 0.07 | 1.6 (0.9, 2.5) | | 0.9 | 2.2 (1.3, 3.8) | | 0.8 |
|  | DM Yes | 1.8 (1.4, 2.5) |  | 1.7 (1.2, 2.4) | |  | 1.7 (1.2, 2.5) | |  |
| **sTNFR1** | Age <74 | 3.4 (2.4, 4.9) | 0.41 | 1.8 (1.0, 3.0) | | 0.23 | 2.7 (1.8, 4.1) | | 0.017 |
|  | Age ≥74 | 2.4 (1.6, 3.5) |  | 2.5 (1.5, 4.1) | |  | 1.8 (1.0, 3.3) | |  |
| **sTNFR2** | Age <74 | 2.4 (1.8, 3.3) | 0.80 | 1.9 (1.2, 2.9) | | 0.48 | 2.1 (1.4, 3.2) | | 0.06 |
|  | Age ≥74 | 2.2 (1.5, 3.3) |  | 2.1 (1.3, 3.6) | |  | 1.9 (1.0, 3.6) | |  |
| **KIM-1** | Age <74 | 2.1 (1.6, 2.7) | 0.55 | 1.7 (1.2, 2.3) | | 0.22 | 1.8 (1.3, 2.4) | | 0.81 |
|  | Age ≥74 | 2.0 (1.4, 2.7) |  | 1.8 (1.2, 2.9) | |  | 2.0 (1.2, 3.1) | |  |
| **sTNFR1** | eGFR <60 | 3.5 (2.3, 5.3) | 0.39 | 2.8 (1.6, 4.7) | | 0.39 | 6.3 (2.6, 14.8) | | 0.20 |
|  | eGFR ≥60 | 2.5 (1.7, 3.5) |  | 1.8 (1.1, 2.9) | |  | 2.7 (1.9, 4.0) | |  |
| **sTNFR2** | eGFR <60 | 2.5 (1.7, 3.7) | 0.51 | 2.3 (1.5, 3.6) | | 0.26 | 3.5 (1.6, 7.6) | | 0.97 |
|  | eGFR ≥60 | 2.1 (1.6, 2.8) |  | 1.6 (1.0, 2.4) | |  | 2.4 (1.7, 3.4) | |  |
| **KIM-1** | eGFR <60 | 1.8 (1.3, 2.4) | 0.0048 | 1.7 (1.2, 2.4) | | 0.43 | 1.7 (0.9, 3.1) | | 0.045 |
|  | eGFR ≥60 | 2.7 (2.0, 3.6) |  | 1.9 (1.3, 2.8) | |  | 2.7 (2.0, 3.8) | |  |

*Adjusted for recipient Age (per year), recipient gender, white race (Yes/No), non-elective surgery, surgery type, pre-op eGFR, diabetes, hypertension, CHF, history of MI, pre-op urine albumin and urine creatinine, clinical site

**Supplementary Table 4.** Pre-operative patient characteristics stratified by availability of CKD outcome assessment in follow-up

|  | **CKD outcome available** | |
| --- | --- | --- |
| **Characteristic** | No (n=556) | Yes (n=837) |
| Age, mean (at time of surgery) | 71.9 ± 10.8 | 71.8 ± 8.9 |
| White | 503 (90.5%) | 809 (96.7%) |
| Male | 370 (66.5%) | 593 (70.8%) |
| Diabetes | 202 (36.3%) | 337 (40.3%) |
| Hypertension | 452 (81.3%) | 660 (78.9%) |
| EF <35% or grade 3 or 4 LV dysfunction | 71 (12.8%) | 65 (7.8%) |
| Myocardial infarction | 146 (26.3%) | 211 (25.2%) |
| Congestive heart failure | 209 (37.6%) | 105 (12.5%) |
| Mean Preoperative serum Cr, mg/dL | 1.1 ± 0.4 | 1.0 ± 0.3 |
| Mean Preoperative eGFR, mL/min/1.73m^2^ | 65.8 ± 20.3 | 69.8 ± 18.3 |
| CKD stage ≥ 3 | 215 (38.7%) | 250 (29.9%) |
| Mean Urine Albumin to Cr ratio, mg/g | 0.2 ± 2.2 | 0.1 ± 0.2 |
| Surgery type |  |  |
| CABG and valve | 125 (22.5%) | 185 (22.1%) |
| Other | 10 (1.8%) | 9 (1.0%) |
| CABG only | 263 (47.3%) | 416 (49.7%) |
| Valve only | 158 (28.4%) | 227 (27.1%) |
| Nonelective surgery | 400 (71.9%) | 763 (91.2%) |
